# Preventive effect of insect tea against reserpine-induced gastric ulcers in mice

**DOI:** 10.3892/etm.2014.1859

**Published:** 2014-07-23

**Authors:** YA-LIN ZHOU, RUI WANG, XIA FENG, XIN ZHAO

**Affiliations:** Department of Biological and Chemical Engineering, Chongqing University of Education, Nan’an, Chongqing 400067, P.R. China

**Keywords:** insect tea, gastric ulcer, cytokine, ICR mice, quantitative polymerase chain reaction

## Abstract

The aim of the present study was to determine the preventive effect of insect tea against reserpine-induced gastric ulcers in ICR mice. A high (800 mg/kg) dose of insect tea reduced the serum levels of the proinflammatory cytokines interleukin (IL)-6, IL-12, tumor necrosis factor (TNF)-α and interferon (IFN)-γ compared with those in mice treated with a low (400 mg/kg) dose and the control mice. The serum levels of somatostatin (SS) and vasoactive intestinal peptide (VIP) in mice treated with insect tea were higher compared with those in the control mice; however, the serum levels of motilin (MOT) and substance P (SP) were lower in mice treated with insect tea than in the control mice. Gastric ulcer inhibitory rate of the insect tea treatment group of mice were much lower compared to the control mice, and the high concentration treated mice were similar to the ranitidine treated mice. The activities of superoxide dismutase (SOD) and glutathione peroxidase (GSH-Px) in mice treated with insect tea were higher compared with those in control mice, and similar to those in normal mice and ranitidine-treated mice. The nitric oxide (NO) and maleic dialdehyde (MDA) levels of mice treated with a high concentration of insect tea compared with the normal group were close. Using quantitative polymerase chain reaction (qPCR) assays, the present study revealed that insect tea significantly induced inflammation in the tissues of mice by downregulating the expression of nuclear factor κ-light-chain-enhancer of activated B cells (NF-κB), inducible nitric oxide synthase (iNOS) and cyclooxygenase (COX)-2, and upregulating the expression of nuclear factor of κ light polypeptide gene enhancer in B-cells inhibitor α (IκB-α). These results suggest that insect tea is as effective at preventing gastric ulcers as the gastric ulcer drug, ranitidine and it can be used as medicine.

## Introduction

Insect tea (tea produced from insect droppings) is a natural organic tea that is based on the essences of animals and plants. Records exist from as early as the Ming Dynasty (1368–1644) of its positive effects on nutrition and health (1). Local residents placed wild rattan and leaves of the Kuding tea plant (*Ilex kudingcha* C.J. Tseng), vine tea plant (*Ampelopsis megalophylla* Diels et Gilg), dyetree (*Platycarya strobilacea* Sieb. et Zucc) and Toringo [*Malus sieboldii* (Regel) Rehd] together to lure the larvae of *Hydrillodes morosa* (Butler), *Nodaria niphona* (Butler), *Aglossa dimidiata* (Haworth), *Herculia glaucinalis* and *Fujimacia bicoloralis* (Leech). The larvae subsequently consumed and excreted the leaves. The residue of rattan and tea leaves was extracted from the droppings (locally named as ‘dragon balls’) and baked to prepare the tea (2).

Gastric ulcers commonly affect the gastro-intestinal tract and cause inflammatory injuries in the gastric mucosa (3). Reserpine is an indole alkaloid drug; however, due to its numerous side-effects, it is rarely used as a medicine (4). Reserpine has a peripheral action with a number of effects on the cholinergic part of the autonomic nervous system that controls the gastro-intestinal tract and smooth muscles (5). Reserpine may cause gastric intolerance, gastric ulcers, stomach cramps and diarrhea (6). Thus, based on the methods of a previous study (7), reserpine was used to induce gastric ulcers in the current study.

In the present study, the preventive effect of insect tea on gastric ulcers was investigated. The serum levels of the inflammation-related cytokines motilin (MOT), somatostatin (SS), substance P (SP) and vasoactive intestinal peptide (VIP) were used to evaluate the preventive effects of insect tea against reserpine-induced gastric ulcers in ICR mice. Gastric tissue histology was also used to determine the preventive effects *in vivo*. The levels of superoxide dismutase (SOD), glutathione peroxidase (GSH-Px), nitric oxide (NO) and maleic dialdehyde (MDA) in the mouse tissues were tested, and certain mRNA gene expression levels in the tissues were also determined.

## Materials and methods

### Preparation of insect tea

Insect tea was purchased from Chishui Green Ecology Industry Company (Chishui, China). The tea was stored at −80°C and freeze-dried to produce a powder. A ten-fold volume of boiling water was added to the powdered sample and extraction was conducted twice. The aqueous extract was evaporated using a rotary evaporator (N-1100; Eyela, Tokyo, Japan) in order to concentrate it.

### Animals

Seven-week-old male ICR mice (n=50) were purchased from the Experimental Animal Center of Chongqing Medical University (Chongqing, China). They were maintained in a temperature-controlled facility (temperature 23±1°C, relative humidity 50±5%) with a 12 h light/dark cycle. The mice had unlimited access to a standard mouse chow diet and water.

### Gastric ulcer experiment

The mice were divided into five groups (n=10 each). Mice in the normal group received no treatment during the experimental period. The control group mice received no treatment for the first four weeks. The mice in the low and high dose insect tea groups received an oral administration of 400 and 800 mg/kg, respectively, of insect tea every day for four weeks. The drug-cure group mice received a 50 mg/kg oral dose of ranitidine for four weeks as a positive control. Then, the control, low dose insect tea, high dose insect tea and ranitidine groups were administered single intraperitoneal injections of 10 mg/kg body weight/day reserpine (Sigma, St. Louis, MO, USA) for three days. Following the final injection, all mice underwent fasting for 24 h; the stomachs were then removed, inflated by injecting 10 ml 1% formalin for 10 min to fix the tissue walls and opened along the greater curvature (8). Hemorrhagic lesions that developed in the stomach (used to assess the gastric ulcer inhibition rate) were measured using a digital camera (D550; Canon, Tokyo, Japan) with a square grid and the images were analyzed using ImageJ software (National Institutes of Health, Bethesda, MD, USA). The gastric ulcer inhibitory rate (%) = (gastric ulcer area of control mice - gastric ulcer area of treated mice)/gastric ulcer area of control mice. These experiments followed a protocol that was approved by the Animal Ethics Committee of Chongqing Medical University (Chongqing, China).

### Analysis of the levels of inflammatory-related cytokines in the serum by enzyme-linked immunosorbent assay (ELISA)

For the serum cytokine assay, blood from the inferior vena cava was collected and centrifuged at 825 × g, 4°C for 10 min. The serum was aspirated and assayed as described below. Concentrations of the inflammation-related cytokines interleukin (IL)-6, IL-12, tumor necrosis factor (TNF)-α and interferon (IFN)-γ in the serum were measured by ELISA according to the manufacturer’s instructions (BioLegend, San Diego, CA, USA). Biotinylated antibody reagent and the supernatants of the homogenized serum were added to 96-well plates which were subsequently incubated at 37°C in CO_2_ for 2 h. Following washing with phosphate-buffered saline (PBS), streptavidin-horseradish peroxidase (HRP) solution was added and the plates were incubated for 30 min at room temperature. The absorbance was measured at 450 nm using a microplate reader (iMark; Bio-Rad, Hercules, CA, USA) (9).

### Determination of the serum levels of MOT, somatostatin, substance P and VIP

Blood was collected from the mice and centrifuged at 1,100 × g, 4°C for 10 min. The levels of MOT, SS, SP and VIP in the serum were determined using commercially available kits (Beijing Puer Weiye Biotechnology Co., Ltd., Beijing, China).

### Determination of the levels of SOD, GSH-Px, NO and MDA

The gastric tissues were homogenated using a high-speed tissue homogenizer (T10; IKA^®^-Werke GmbH & Co. KG, Staufen, Germany) at 4,000 rpm, 4°C for 10 min. The contents of SOD, GSH-Px, NO and MDA were determined using commercially available kits (Nanjing Juli Institute of Biomedical Engineering, Nanjing, China).

### mRNA expression levels

The total RNA from gastric tissue cells was isolated using TRIzol reagent (Invitrogen Life Technologies, Carlsbad, CA, USA) according to the manufacturer’s recommendations. The RNA was digested with RNase-free DNase (Roche Diagnostics, Basel, Switzerland) for 15 min at 37°C and purified using an RNeasy kit (Qiagen, Hilden, Germany) according to the manufacturer’s instructions. The cDNA was synthesized from 2 μg total RNA by incubation at 37°C for l h with avian myeloblastosis virus reverse transcriptase (GE Healthcare, Little Chalfont, United Kingdom) with random hexanucleotides according to the manufacturer’s instructions. The sequences of the primers used to specifically amplify the genes of interest were as follows: nuclear factor κ-light-chain-enhancer of activated B cells (NF-κB), 5′-CAC TTA TGG ACA ACT ATG AGG TCT CTG G-3′ (forward) and 5′-CTG TCT TGT GGA CAA CGC AGT GGA ATT TTA GG-3′ (reverse); nuclear factor of κ light polypeptide gene enhancer in B-cells inhibitor, α (IκB-α), 5′-GCT GAA GAA GGA GCG GCT ACT-3′ (forward) and 5′-TCG TAC TCC TCG TCT TTC ATG GA-3′ (reverse); inducible nitric oxide synthase (iNOS), 5′-AGA GAG ATC GGG TTC ACA-3′ (forward) and 5′-CAC AGA ACT GAG GGT ACA-3′ (reverse); and cyclooxygenase (COX)-2, 5′-TTA AAA TGA GAT TGT CCG AA-3′ (forward) and 5′-AGA TCA CCT CTG CCT GAG TA-3′ (reverse). Glyceraldehyde 3-phosphate dehydrogenase (GAPDH) was amplified as an internal control gene with the following primers: 5′-CGG AGT CAA CGG ATT TGG TC-3′ (forward) and 5′-AGC CTT CTC CAT GGT CGT GA-3′ (reverse). Amplification was performed in a thermal cycler (Eppendorf, Wesseling, Germany). The polymerase chain reaction (PCR) products were separated in 1.0% agarose gels and visualized with ethidium bromide staining (10).

### Statistical analysis

Data are presented as mean± standard deviation. Differences between the mean values for individual groups were assessed using a one-way analysis of variance (ANOVA) with Duncan’s multiple range test. P<0.05 was considered to indicate a statistically significant difference. SAS version 9.1 (SAS Institute Inc., Cary, NC, USA) was used for statistical analyses.

## Results

### Gastric ulcer inhibitory effects

**Figure 1:**
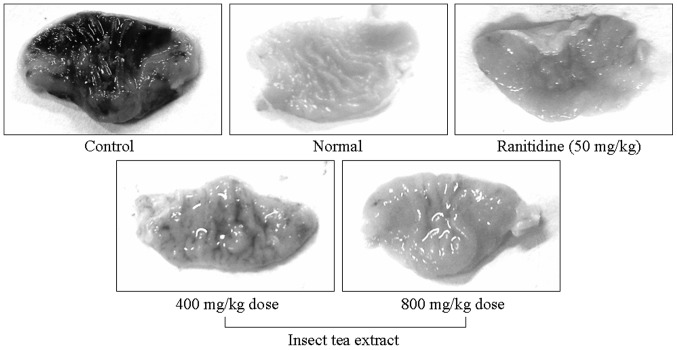
Stomachs of the mice pretreated with insect tea and then subjected to the induction of gastric ulcers with reserpine.

The administration of insect tea to mice prior to the induction of gastritis led to a reduced presence of gastric ulcers. The mice in the control group demonstrated an average gastric ulcer area of 18.92±3.41 mm^2^. The mice treated with ranitidine had lower levels of gastric ulcers compared with those in the mice treated with insect tea. Treatment with 400 and 800 mg/kg insect tea resulted in an average gastric ulcer area of 5.68±0.55 mm^2^ (inhibitory rate 69.98%) and 3.98±0.61 mm^2^ (inhibitory rate 78.97%), respectively (Table I and Fig. 1).

### Levels of IL-6, IL-12, TNF-α and IFN-γ in the serum

**Figure 2:**
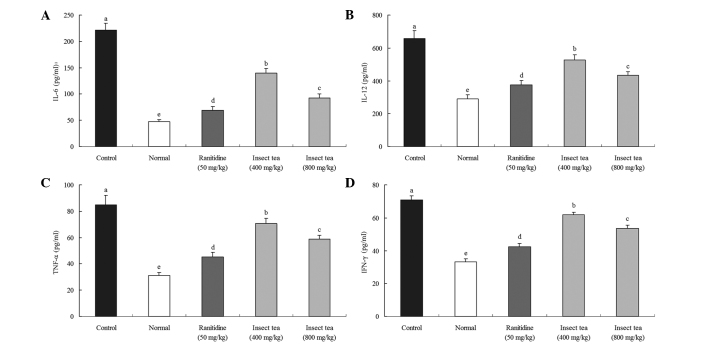
Levels of (A) interleukin (IL)-6, (B) IL-12, (C) tumor necrosis factor α (TNF-α) and (D) interferon γ (IFN-γ) in the serum of mice from different treatment groups with reserpine-induced gastric ulcers. ^a–e^Mean values with different letters over the bars are significantly different (P<0.05) according to Duncan’s multiple range test.

The level of IL-6 in the normal mice was 47.6±3.3 pg/ml; however, in the control mice it was significantly higher at 221.6±13.2 pg/ml (Fig. 2). The levels of IL-6 in mice treated with 400 and 800 mg/kg insect tea extract were 139.7±8.8 and 92.3±7.9 pg/ml, respectively. For the mice treated with ranitidine, the level of IL-6 was 68.7±7.5 pg/ml. The control mice demonstrated the highest level of IL-12 out of all the groups at 657.1±47.2 pg/ml. Ranitidine, and 400 and 800 mg/kg insect tea reduced the levels of IL-12 to 374.5±25.6, 527.9±31.2, 432.1±22.6 pg/ml, respectively; and the normal mice revealed the lowest level at 288.3±27.6 pg/ml (Fig. 2). The levels of TNF-α in the control, normal, ranitidine, and 400 and 800 mg/kg insect tea-treated mice were 84.9±7.1, 31.2±2.2, 45.2±3.6, 70.6±3.8 and 58.7±2.9 pg/ml, respectively (Fig. 2). The serum levels of IFN-γ in the mice treated with 400 (61.8±1.7 pg/ml) and 800 (53.6±2.0 pg/ml) mg/kg insect tea were significantly lower compared with those in the control group (70.7±2.8 pg/ml). The levels of IFN-γ in the normal and ranitidine-treated mice were 33.2±1.8 and 42.3±2.2 pg/ml, respectively (Fig. 2).

### Serum levels of MOT, SS, SP and VIP

**Figure 3:**
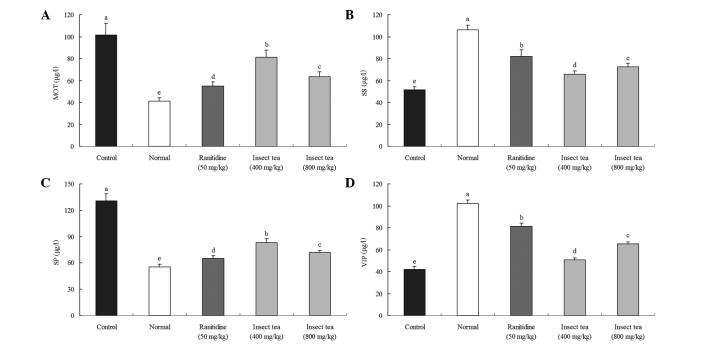
Levels of (A) motilin (MOT), (B) somatostatin (SS), (C) substance P (SP) and (D) vasoactive intestinal peptide (VIP) in the serum of mice from different treatment groups with reserpine-induced gastric ulcers. ^a–e^Mean values with different letters over the bars are significantly different (P<0.05) according to Duncan’s multiple range test.

The level of MOT in the normal mice was 41.3±2.8 μg/l; however, it was significantly higher in the control mice at 101.5±2.8 μg/l (Fig. 3). The levels of MOT in the 400 and 800 mg/kg insect tea groups were 81.2±6.6 and 63.7±4.1 μg/l, respectively. In the mice treated with ranitidine, the level of MOT was 55.1±3.8 μg/l. The level of SS in mice treated with 800 mg/kg insect tea increased to 72.6±3.1 μg/l, which was higher than the level in mice treated with 400 mg/kg insect tea (65.6±3.41 μg/l; Fig. 3). The levels of SS in the control, normal and 50 mg/kg ranitidine-treated mice were 51.6±2.8, 106.3±4.3 and 82.1±6.1 μg/l, respectively. The level of SP in the normal group was 55.6±3.15 μg/l, whereas that of the control group was 130.5±8.3 μg/l, reflecting a marked increase. The SP levels in the 400 and 800 mg/kg insect tea groups decreased from those in the control group to 83.6±3.8 and 72.3±1.8 μg/l, respectively. Ranitidine treatment also resulted in a decreased level of SP (65.2±3.3 μg/l; Fig. 3). The levels of VIP in the 400 and 800 mg/kg insect tea groups were 50.8±1.8 and 65.3±1.9 μg/l, respectively, which were higher than the VIP level in the control group (42.3±2.6 μg/l). However, the level of VIP in the ranitidine-treated group was 81.2±2.98 μg/l and the normal group demonstrated the highest level at 102.3±3.1 μg/l (Fig. 3).

### Content of SOD, GSH-Px, NO and MDA in the gastric tissue

**Figure 4:**
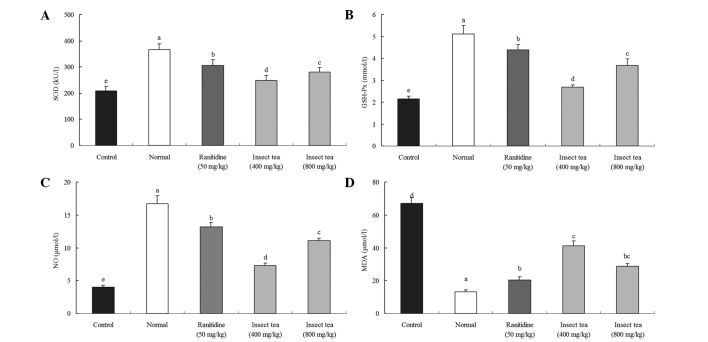
Levels of superoxide dismutase (SOD), glutathione peroxidase (GSH-Px), nitric oxide (NO) and maleic dialdehyde (MDA) in the tissue of mice from different treatment groups with reserpine-induced gastric ulcers. ^a–e^Mean values with different letters over the bars are significantly different (P<0.05) according to Duncan’s multiple range test.

The levels of SOD and GSH-Px in the gastric tissue of the control mice were 208.3±17.6 kU/l and 2.14±0.14 mmol/l, respectively (Fig. 4). In the normal mice, these levels were notably increased at 367.2±21.8 kU/l and 5.12±0.39 mmol/l, respectively. The mice treated with 800 mg/kg insect tea and ranitidine demonstrated similar levels of SOD at 281.1±16.6 and 305.6±22.6 kU/l, whilst the 400 mg/kg insect tea-treated mice had a lower level (247.9±19.4 kU/l). The levels of GSH-Px in the mice treated with ranitidine (4.38±0.25 mmol/l) and 800 mg/kg insect tea (3.67±0.31 mmol/l) were higher compared with those in the mice treated with 400 mg/kg insect tea (2.68±0.12 mmol/l). The levels of NO in the mice of the control, normal, ranitidine, 400 mg/kg and 800 mg/kg insect tea groups were 4.0±0.3, 16.7±1.2, 13.2±0.6, 7.3±0.4 and 11.1±0.4 μmol/l, respectively (Fig. 4). The levels of MDA in these groups revealed the opposite trend at 67.2±3.2, 13.1±1.3, 20.3±2.1, 41.2±3.1 and 28.7±1.8 μmol/l, respectively (Fig. 4).

### Inflammation-related gene expression levels of NF-κB, IκB-α, iNOS and COX-2

**Figure 5:**
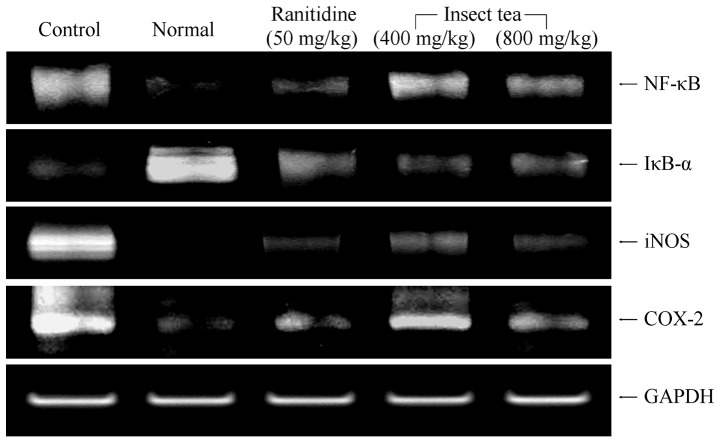
Effect of insect tea on the mRNA expression of nuclear factor κ-light-chain-enhancer of activated B cells (NF-κB), nuclear factor of κ light polypeptide gene enhancer in B-cells inhibitor α (IκB-α), inducible nitric oxide synthase (iNOS) and cyclooxygenase 2 (COX-2) in reserpine-induced gastric ulcers in mice. GADPH, glyceraldehyde 3-phosphate dehydrogenase.

PCR assays were conducted to investigate whether the anti-inflammatory effects of insect tea were associated with the inhibition of the expression of the inflammation-related genes NF-κB, IκB-α, iNOS and COX-2. As shown in Fig. 5, the mRNA expression levels of NF-κB were reduced in the gastric tissues of mice treated with insect tea or ranitidine compared with those in the control group. Insect tea and ranitidine significantly modulated the expression level of genes associated with inflammation. The mRNA expression level of NF-κB was decreased while the mRNA and protein levels of IκB-α were increased compared with those in the control group. Furthermore, the mRNA expression levels of COX-2 and iNOS were decreased by treatment with insect tea in a dose-dependent manner compared with those in the control group. These results indicate that insect tea may help to prevent gastric ulcers by increasing anti-inflammatory activities. Overall, the results of the present study indicated that insect tea had a strong anti-inflammatory effect against gastric ulcers.

## Discussion

Although insect tea has been used as a traditional healing drink for centuries, little scientific data is available concerning its medical effects. Insect tea contains large amounts of flavonoids, insect hormones, prothrombin, essential amino acids and trace elements, particularly Fe, Zn, Ca and Mg, all of which are present at higher levels than are found in other types of tea. Insect tea also contains a number of other nutrients, including crude protein, crude fiber, fat, polyphenols, caffeine, sugar and vitamins (11,12). Insect tea has been reported to have various functional effects, including antipyretic and hemostatic effects, and the ability to prevent high blood pressure, hyperlipidemia and coronary heart disease (1). In the present study, the anti-gastric ulcer effects of insect tea were investigated. From the results of animal experiments, insect tea demonstrated a strong preventive effect on reserpine-induced gastric ulcers.

The serum levels of cytokines, including IL-6, IL-12 and TNF-α, are higher in patients with inflammatory diseases than in healthy individuals (13). Cytokine receptors and the inflammatory cytokines IL-6, IL-12, TNF-α and IFN-γ play a pathogenic role in gastric disease. Thus, lower levels of these cytokines are indicative of an improved gastric ulcer preventive effect (14,15). IL-6 is an interleukin that functions as a proinflammatory and anti-inflammatory cytokine, and is encoded by the IL6 gene in humans (16). IL-6 is secreted by T cells and macrophages to initiate an immune response, in particular when damage occurs to a tissue, leading to inflammation. IL-6 is also involved in fighting infection (17). IL-12 contributes to inflammation eradication through the IFN-γ-dependent induction of the antiangiogenic factors interferon-inducible protein (IP) 10 and monokine induced by γ interferon (MIG) (18). TNF-α is a cytokine involved in systemic inflammation, and is a member of a group of cytokines that stimulate the acute phase reaction (19). Inflammatory cytokines, IL-6 and TNF-α, play a pathogenic role in diseases of the stomach (20). Traumatic hemorrhage in the stomach causes the levels of systemic IL-6 to increase; however, impairment of hepatocellular function occurs and gastric injury may develop (21). In the present study, the serum levels IL-6, IL-12, TNF-α, and IFN-γ in the mice with reserpine-induced gastric ulcers were markedly decreased following treatment with insect tea. Based on the results of the current study, insect tea is an effective preventive treatment for gastric ulcers, and a high dose of insect tea may further enhance its preventive effects.

MOT and SP are excitatory gastrointestinal hormones and their content increases following stimulation (22). Upon stimulation, MOT causes a surplus secretion of gastric acid. An excess of gastric acid causes the inner part of the stomach to become acidic, thus compounding the gastric ulcers (23). In the present study, the levels of MOT and SP increased due to the inductive effect of reserpine. SS and VIP are inhibitive gastrointestinal hormones that are able to inhibit the secretion of gastric acid (22). It has been demonstrated that injury of the gastric mucosa results in the surplus secretion of gastric juice, which causes the gastric pH value to be lower than the normal value (24). In the present study, the levels of SS and VIP were increased following treatment with a high dose of insect tea compared with those in the control mice. These changes may be expected to lead to a reduction in the volume of gastric secretions and an increase in the pH of the gastric juice.

Following the deterioration of gastric tissue due to the presence of gastric ulcers, parts of the tissues may become oxidized due to the damage induced. As important antioxidases, SOD and GSH-Px reduce peroxides to harmless or less harmful substances, which is beneficial to recovery from gastric ulcers (25). Gastric ulcers are caused by an imbalance between the damage experienced by gastric tissue and the presence of protective factors. NO serves to protect the gastric mucosa and maintain a smooth blood flow. Levels of NO markedly decrease in patients suffering from gastric ulcers, and NO has been demonstrated to be an effective component in the prevention of gastric ulcers (26). MDA is a marker of oxidative stress and is generated in large amounts in damaged areas of gastric tissue. Therefore, it may be regarded as an indicator of gastric ulceration (27). The results of the present study demonstrated that a higher dose of insect tea decreased the prevalence of gastric ulcers.

NF-κB, IκB-α, iNOS and COX-2 genes in the tissue may be used as potential biomarkers to monitor damage to the viscera. NF-κB is a ubiquitous transcription factor that regulates the expression of genes required for cellular proliferation, inflammatory responses and cell adhesion (28). IκB α is also an inflammation related gene, it can release NF-κB to increase inflammation (9,29). Following inflammatory stimulation, COX-2 and iNOS have been reported to induce adverse effects in the stomach (30). The content of iNOS and COX-2 could increase in serum and tissue and could also increase inflammatory responses in early stages (31).

In summary, the preventive effect of insect tea on gastric ulcers was evaluated in the current study through an *in vivo* experiment. Analyses of the stomachs of mice from various treatment groups revealed that insect tea prevented reserpine-induced abdominal ulcers. This indicates that insect tea represents a potentially useful agent for the treatment or prevention of drug-induced gastric ulcers *in vivo*. Following gastric ulcer induction in mice, IκB α released NF-κB at a faster rate and as a result NF-κB gene expression was increased and IκB α expression decreased. Additionally, insect tea was capable of alleviating these changes and thus it was thought to have anti-inflammatory effects.

## Figures and Tables

**Table I:** Prevention of reserpine-induced gastric ulcers in ICR mice by treatment with insect tea.

	Gastric ulceration	
	
Group	Gastric ulcer area (mm^2^)	Inhibitory rate (%)
Control	18.92±3.41^a^	100.00
Normal	0.00±0.00^e^	0.0
Ranitidine (50 mg/kg dose)	2.51±0.74^d^	86.73
Insect tea (400 mg/kg dose)	5.68±0.55^b^	69.98
Insect tea (800 mg/kg dose)	3.98±0.61^c^	78.97

a–e Mean values with different letters in the same column are significantly different (P<0.05) according to Duncan’s multiple range test.
